# Crystal structures of *Escherichia coli* glucokinase and insights into phosphate binding

**DOI:** 10.1107/S2053230X25005515

**Published:** 2025-07-09

**Authors:** Joseph Andrews, Joshua Sakon, Chenguang Fan

**Affiliations:** a Department of Chemistry and Biochemistry University of Arkansas at Fayetteville Fayetteville AR72701 USA; b Cell and Molecular Biology Program University of Arkansas at Fayetteville Fayetteville AR72701 USA; Centre for Cellular and Molecular Biology, Hyderabad, India

**Keywords:** glucokinases, hexokinases, phosphate binding, sulfate binding

## Abstract

Here, we report the crystal structure of *Escherichia coli* glucokinase (GLK), which has phosphate bound in the cleft between the α and β domains adjacent to the active site. A ternary complex consisting of GLK, glucose and phosphate is also reported in this work. Diffraction data were collected at 2.63 Å resolution for the phospate-bound form (*R*_work_/*R*_free_ = 0.191/0.230) and at 2.54 Å resolution for the ternary complex (*R*_work_/*R*_free_ = 0.202/0.258), both at 297 K. A *B*-factor analysis of the phosphate-bound GLK structure revealed consistently lower values for phosphate-interacting basic residues in the α4, α5 and α9 helices, while significant root-mean-square deviation (r.m.s.d.) spikes indicated flexibility in regions preceding β1 and within the loop between the β5 and β6 sheets of the α domain. In the ternary complex, phosphate is bound adjacent to glucose, and the *B* factors for the α4, α5 and α9 helices were further reduced, while r.m.s.d. spikes were observed at the end of the β10 sheet and within the α6 helix of the β-domain. This structural characterization suggests that phosphate could influence the activity of GLK by altering glucose binding and modulating interactions with a loop-interacting regulatory protein.

## Abstract 2

This work presents the structure of apo *Escherichia coli* glucokinase (ecGLK) bound with sulfate, a phosphate mimic, and the structure of ecGLK in complex with both glucose and phosphate, implying a role for phosphate in regulating the glucokinase activity.

## 1. Introduction

Glucokinase (GLK) catalyzes the first reaction in the glycolytic pathway and is a target for regulation in a wide range of organisms. GLKs are divided into three families based on sequence identities. Group I GLKs comprise ADP- and ATP-dependent GLKs from archaea and a small selection of eukaryotes (Sakuraba *et al.*, 2004 ▸; Ronimus & Morgan, 2004 ▸). Group II GLKs are ATP-dependent enzymes that lack the classical repressor open reading frame kinase (ROK) sequence motif and contain more than 50 partial and complete protein sequences, including *Escherichia coli* GLK (ecGLK; Titgemeyer *et al.*, 1994 ▸; Meyer *et al.*, 1997 ▸). Group III GLKs comprise ATP-dependent GLKs from bacteria and archaea which possess the ROK sequence motif (Hansen *et al.*, 2002 ▸; Hansen & Schönheit, 2003 ▸; Scorpio *et al.*, 2004 ▸).

In a previous structural study, apo ecGLK crystals were obtained using the vapor-diffusion method (Lunin *et al.*, 2004 ▸). The resulting crystal unit-cell dimensions are *a* = *b* = 81.5, *c* = 234.7 Å in space group *P*4_3_2_1_2, which includes two molecules in the asymmetric unit. EcGLK–glucose complex crystals were also produced using the vapor-diffusion method with 2 m*M* glucose. These ecGLK–glucose crystals exhibited unit-cell dimensions *a* = 78.416, *b* = 53.538, *c* = 90.903 Å in space group *P*12_1_1, also containing two molecules in the asymmetric unit. This work showed that during the ecGLK binding-site transformation, the maximum C^α^ displacement of residues in the small domain on superposing two apo ecGLK monomers was observed at Thr78, indicating that this portion of the loop closes during glucose binding. Superposition of the small domain revealed 320 C^α^ atoms with a root-mean-square deviation (r.m.s.d.) of 1.3 Å. In comparison, the large domain consisted of 186 C^α^ atoms with an r.m.s.d. of 0.38 Å. The binding of glucose was proposed to stabilize the flexible loops of ecGLK, allowing the monomers to adopt a similar conformation. In the absence of glucose, apo ecGLK also demonstrates intrinsic flexibility. During the closure of GLK, residues 73–79 undergo movement, resulting in broken hydrogen bonds between the small and large domains (between Arg16 NE and Thr32 OG1 and between Trp131 O and Glu315 NE2). The closure of the domains generates new hydrogen bonds between Arg16 NE and Asn303 OD, along with Arg16 NH1 and Thr32 OG (Lunin *et al.*, 2004 ▸).

To date, there are no ecGLK structures reported together with the other substrate ATP or the product ADP. This work presents the structure of apo ecGLK bound with phosphate and the structure of ecGLK in complex with both glucose and phosphate, implying a role for phosphate in regulation of the GLK activity.

## 2. Materials and methods

### 2.1. Purification and crystallization

Tbe expression and purification of ecGLK followed our previous work on this enzyme (Fatema *et al.*, 2024 ▸). Briefly, the *E. coli glk* gene was cloned into the pCDF-1b plasmid with a His_6_-tag at the C-terminus and was transformed into *E. coli* BL21 (DE3) cells. The expression strain was grown in 400 ml LB medium with 100 µg ml^−1^ streptomycin at 37°C to an absorbance of between 0.6 and 0.8 at 600 nm. Expression of ecGLK was induced by adding 0.1 m*M* isopropyl β-d-1-thiogalactopyranoside (IPTG) and incubating at 16°C for an additional 16 h. Cells were harvested and centrifuged at 3000*g* for 20 min. Cell pellets were stored at −80°C. The purification process involved several steps, including the addition of 12 ml lysis buffer (Tris–HCl pH 7.5, 300 m*M* NaCl, 20 m*M* imidazole) to the cell pellet along with 5 µl β-mercaptoethanol, protease inhibitors and nuclease. The mixture was sonicated for 3 min on ice and then centrifuged at 19 000*g* for 25 min. The supernatant was collected and then filtered using a 0.45 nm filter. The filtered supernatant was added to an affinity chromatography column that contained 2 ml Ni–NTA resin previously equilibrated with 20 ml lysis buffer. The loaded column was washed with 15 ml Tris–HCl pH 7.5 with 50 m*M* imidazole and 300 m*M* NaCl, and 2 ml fractions were then eluted using Tris–HCl pH 7.5 with 200 m*M* imidazole and 300 m*M* NaCl. SDS–PAGE was used to verify the purity of the ecGLK enzyme. The elution fractions were then added to a PD10 column for desalting using desalting buffer (5 m*M* Na_2_HPO_4_, 1 m*M* NaH_2_PO_4_ pH 7.5, 10 m*M* NaCl, 1 m*M* DTT). The sample was concentrated using a protein concentrator with a cutoff of 100  000 at 3000*g* until the concentration reached 7–8 mg ml^−1^. Before crystallization, ecGLK was incubated with 5 m*M* glucose for 1 h. Crystal screening was initiated using Rigaku Berkeley Screen solutions 1–96. The sitting-drop vapor-diffusion method was used with two drops: one containing 0.7 µl protein and 1 µl well solution and the second containing 0.5 µl protein and 1 µl well solution. All of the drops were suspended over 35 µl well solution at 293 K. The initial crystallization conditions were refined by systematically altering the buffer, pH and PEG concentrations until diffraction-quality crystals were grown. All the experiments retained the original protein and well solution volume ratio.

### 2.2. Data collection and processing

Single protein crystals were mounted on a MicroRT-compatible loop holder with a nylon loop (MiTeGen) and then sealed using a MicroRT capillary tube. At the tip of the capillary tube 5 µl mother liquor was present, and the bottom was sealed with vacuum grease. The crystals were sealed at room temperature and the data were collected at room temperature. X-ray diffraction data were collected using a Rigaku XtaLAB Synergy-S Diffractometer (University of Arkansas, Fayetteville, USA) and a HyPix-6000HE detector. Raw data images were processed using *CrysAlis^Pro^* and scaled and merged with *AIMLESS*. The structures were determined by the molecular-replacement method using *Phaser* (McCoy *et al.*, 2007 ▸) with ecGLK (PDB entry 1q18; Lunin *et al.*, 2004 ▸) as the model for the structure of phosphate-bound ecGLK (LLG score 7087.594) and GLK in complex with glucose (PDB entry 1sz2; Lunin *et al.*, 2004 ▸) as the model for the structure of the phosphate-bound glucose–ecGLK complex (LLG score 4707.901). Structures were refined using *AutoBuild* for automated model building after molecular replacement (Terwilliger *et al.*, 2008 ▸) and *phenix.refine* for model refinement after building (Afonine *et al.*, 2012 ▸). The structures were manually adjusted using *WinCoot* (Emsley *et al.*, 2010 ▸) for validation/refinement and were submitted to the PDB. The PDB codes for apo ecGLK with phosphate and the phosphate-bound ecGLK–glucose complex are 9duc and 9dvz, respectively. Diffraction data and structure statistics are presented in Table 1 ▸. The *R*_merge_ values for both structures are relatively high, possibly resulting from the high multiplicity observed in the data sets. High multiplicity can inflate *R*_merge_ values due to the increased number of observations contributing to the averaged intensity of each reflection.

## 3. Results and discussion

### 3.1. Apo ecGLK versus phosphate-bound ecGLK: effect of phosphate binding

**Figure 1:**
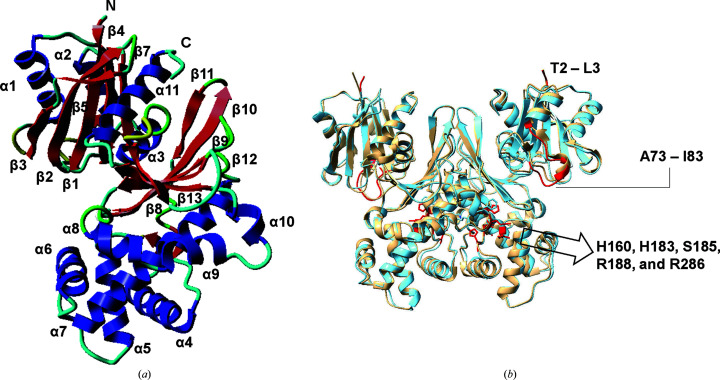
(*a*) Secondary-structure mapping of the ecGLK monomer. The model uses a blue color for α-helices and a red color for β-sheets. β6 is hidden behind because the model is placed in the same direction as in (*b*) for easy identification. (*b*) Comparison of the structures of phosphate-bound ecGLK (PDB entry 9duc, cyan) and apo ecGLK (PDB entry 1q18, tan). Sections with the highest observed differences from the *B*-factor graphing and the r.m.s.d. graphing are highlighted in red.

The phosphate-bound ecGLK crystals were grown in 100 m*M* Tris–HCl pH 8.5, 200 m*M* lithium sulfate, 20%(*w*/*v*) PEG 5000 MME. The phosphate-bound ecGLK structure from the *P*4_3_2_1_2 crystal form was determined by molecular replacement and refined to an *R* factor of 0.1930 (*R*_free_ = 0.2302) at a resolution of 2.65 Å. The phosphate-bound complex is composed of a dimer, with each monomer featuring an α/β domain characterized by noncontiguous segments: the small domain (residues 2–110 and 300–321) and the large domain (residues 111–299). The small domain features β-sheets β1–β4 and β7, as well as α-helices α1–α3 and α11. The small domain α3 helix consisting of residues 100–110 links the small and large domains. In contrast, the large domain features β-sheets β8–β13 and α-helices α4–α10. The dimer interface consists of helix α4 and nearby loops, the C-terminal end of helix α7, strand β10 and the loop that connects strand β10 to strand β11. The phosphate-binding site is found on the edge of the small and large domain cleft outside the α4–α10 cluster. Comparison between phosphate-bound (PDB entry 9duc) and apo (PDB entry 1q18) ecGLK highlights key structural shifts, including His160 and Arg286 (Fig. 1 ▸).

**Figure 2:**
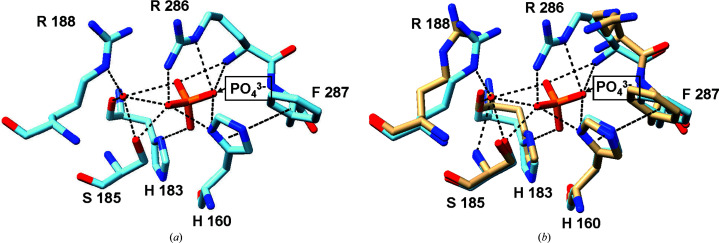
The structure of the phosphate-binding site. (*a*) Amino-acid residues in the phosphate-binding site. (*b*) Superimposition of the residues in apo-form ecGLK (PDB entry 1q18, tan) and phosphate-bound ecGLK (PDB entry 9duc, cyan) at the phosphate-binding site.

His160 in chain *B* experiences conformational changes from the N atom in its imidazole ring (His160 ND1) forming a hydrogen bond to the O atoms (O1 and O4) of the phosphate (Fig. 2 ▸). This bond helps to anchor His160 in the active site, while a π-stacking interaction with Phe287 from chain *A* further reinforces this stability. In contrast, His160 is more flexible in the apo form, indicating that binding to phosphate limits its movement. Arg286 is also important for stabilization through phosphate interaction and undergoes significant structural changes upon binding. The guanidinium group of Arg286 moves closer to the phosphate, allowing the formation of several stabilizing hydrogen bonds. Specifically, Arg286 interacts with O1 and O3 of phosphate in chain *A* through its nitrogen (Arg286 N), and its NH2 group forms hydrogen bonds to O4 of phosphate. Additional interactions between Arg286 NE and phosphate O1 further reinforce this stabilization. In chain *B*, Arg286 similarly forms hydrogen bonds to O1 and O3 of phosphate and Glu187 OE1, indicating its key role in anchoring the phosphate (Fig. 2 ▸).

Besides His160 and Arg286, other amino acids also contribute significantly to phosphate interactions. His183, Ser185 and Glu187 exhibit distinct conformational changes upon phosphate binding. His183 moves closer to phosphate, forming a hydrogen bond to phosphate O4 in chain *A* and phosphate O2 in chain *B*. Ser185 OG establishes a hydrogen bond with phosphate O3. Meanwhile, its backbone interacts with water molecules to maintain stability. Additionally, Glu187 adjusts its position to interact with Arg286 NH2, losing prior interactions with water. Although Arg188 does not directly interact with phosphate, it plays a supporting role by binding water molecules, forming hydrogen bonds that further stabilize the phosphate-bound complex. All of the abovementioned interactions emphasize the effect of phosphate on the structure and stability of ecGLK. The importance of phosphate binding is evident as it helps maintain structural integrity, enhances hydrogen-bonding networks and could impact the catalytic activity.

### 3.2. Glucose–ecGLK versus phosphate-bound glucose–ecGLK: structural changes and functional implications

The crystals of the phosphate-bound glucose–ecGLK complex were obtained by soaking ecGLK with 5 m*M* glucose and were grown in 100 m*M* bis-Tris pH 6.5, 200 m*M* magnesium chloride, 25%(*w*/*v*) PEG 3350. The crystal structure was determined by molecular replacement and refined to an *R* factor of 0.2034 (*R*_free_ = 0.2580) at a resolution of 2.54 Å. The small and large domains retain the α-helices and β-sheets found in phosphate-bound ecGLK as reported above, while there are domain shifts to account for the glucose binding. The small domain shifts down, closing the binding cleft, and the large domain maintains its relative position with slight shifts into the center of ecGLK.

**Figure 3:**
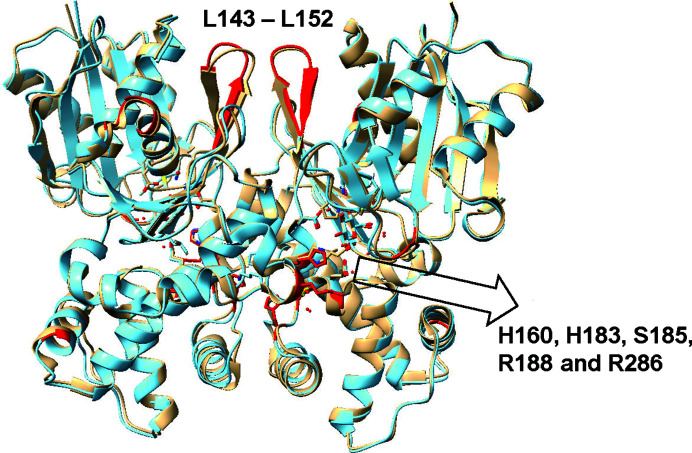
Comparison of the structures of the phosphate-bound glucose–ecGLK complex (PDB entry 9dvz, cyan) and the glucose–GLK complex (PDB entry 1q18, tan). Sections with the highest observed differences from the *B*-factor graphing and the r.m.s.d. graphing are highlighted in red.

The phosphate-bound glucose–ecGLK complex crystallizes in a more highly ordered space group (*P*2_1_2_1_2_1_) compared with the phosphate-free form (*P*12_1_1), suggesting that phosphate binding stabilizes the conformation of ecGLK. When comparing the structure of the glucose–ecGLK complex (PDB entry 1sz2) with the phosphate-bound glucose–ecGLK complex (PDB entry 9dvz), we noticed significant differences, especially in loop flexibility and stabilization of the domains (Fig. 3 ▸). Residues 108–112 (α3–β8 loop) and 143–152 (β10–loop–β11) exhibit notable differences, reflecting conformational shifts upon phosphate binding. Additional structural changes occur in residues 165–173 (loop–α4 helix), 178–182 (α4 helix) and 220–227 (α6–loop–α7). Residues 313–320 at the tip of the large domain also experience structural divergence, further distinguishing the phosphate-bound glucose–ecGLK complex from the phosphate-free form.

**Figure 4:**
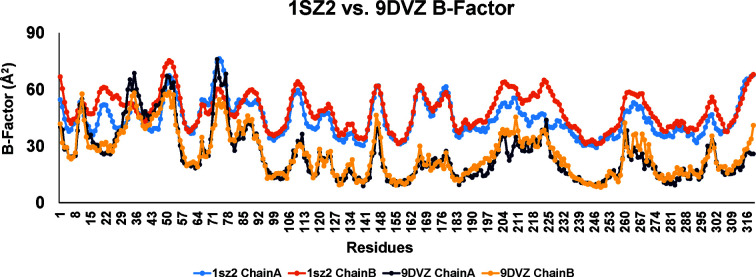
The *B* factors of the structures of the glucose–ecGLK complex (PDB entry 1sz2) and the phosphate-bound glucose–ecGLK complex (PDB entry 9dvz).

A comparison of *B* factors shows a noticeable decrease in structural flexibility when phosphate binds (Fig. 4 ▸). In the glucose–ecGLK complex (PDB entry 1sz2) the average *B* factor is 47.33 Å^2^. It decreases to 27.19 Å^2^ in the phosphate-bound glucose–ecGLK complex (PDB entry 9dvz), suggesting a more rigid structure. Significant differences exist in residues 20–30 (in the β2–loop–β3 region), where the interaction changes, possibly due to solvent access. In the glucose–ecGLK complex (PDB entry 1sz2), Cys20 SG forms a hydrogen bond to Asp21 O and water, while in the phosphate-bound glucose–ecGLK complex (PDB entry 9dvz) it retains only the Asp21 O bond. Similarly, Ala23 O in the phosphate-bound glucose–ecGLK complex (PDB entry 9dvz) gains a new hydrogen bond to Arg314 NH2, stabilizing the loop. Water-mediated interactions in the glucose–ecGLK complex (PDB entry 1sz2), such as those involving Ser24 O, Gly25 O and Glu26 O, are altered or lost in the phosphate-bound glucose–ecGLK complex (PDB entry 9dvz), contributing to its lower *B* factor.

### 3.3. Glucose and phosphate interactions: phosphate as a potential modulator

**Figure 5:**
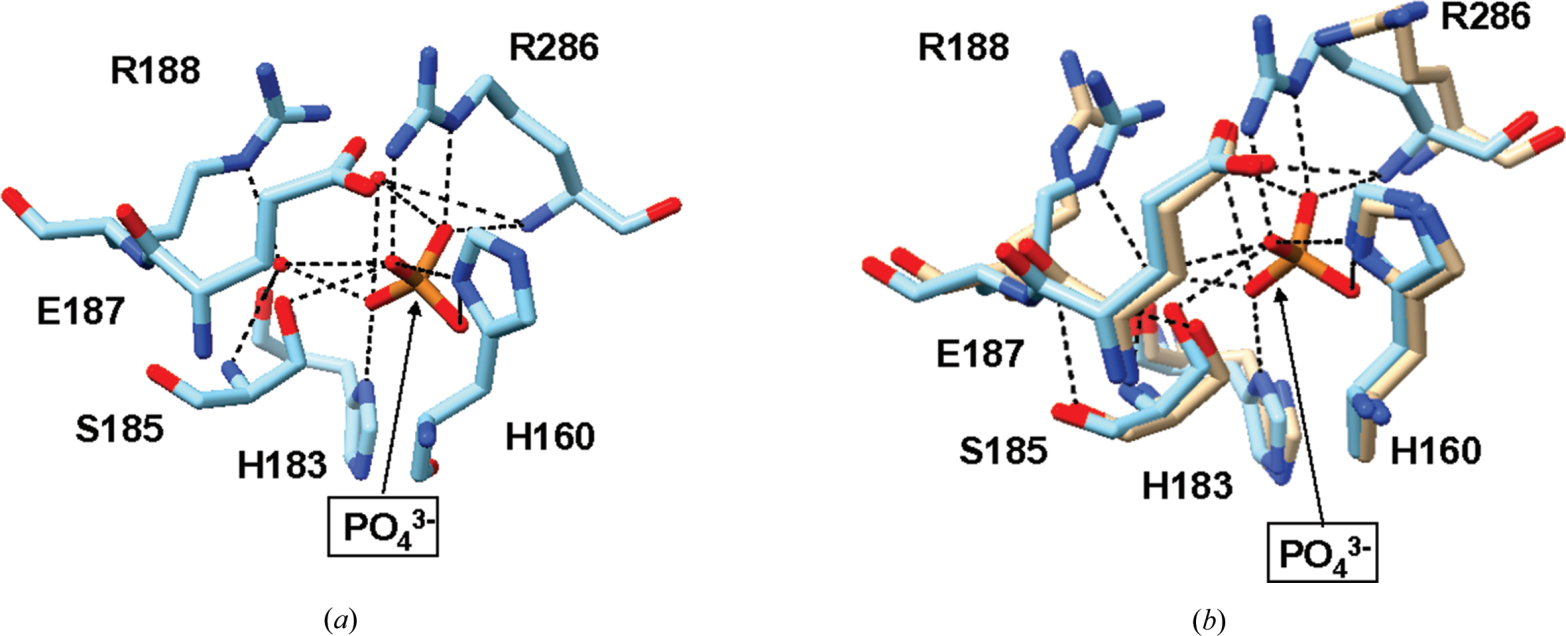
The structure of the phosphate-binding site. (*a*) Amino-acid residues in the phosphate-binding site. (*b*) Superimposition of the residues in the glucose–ecGLK complex (PDB entry 1sz2, tan) and the phosphate-bound glucose–ecGLK complex (PDB entry 9dvz, cyan) at the phosphate-binding site.

**Figure 6:**
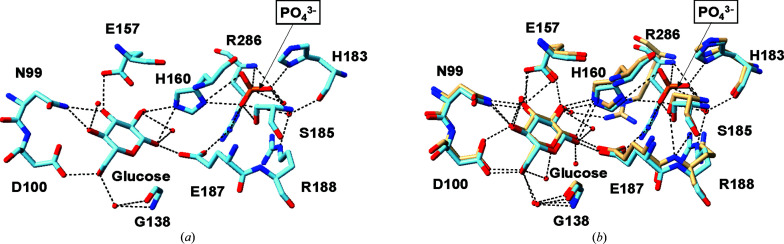
The structure of the glucose- and phosphate-binding sites. (*a*) Amino-acid residues in the glucose- and phosphate-binding sites. (*b*) Superimposition of the residues in the glucose–ecGLK complex (PDB entry 1sz2, tan) and the phosphate-bound glucose–ecGLK complex (PDB entry 9dvz, cyan) at the glucose- and phosphate-binding sites.

When phosphate binds, Arg286 and His160 show conformational changes that affect the stability and hydrogen bonding in the glucose-binding site (Fig. 5 ▸). The phosphate binding stabilizes Arg286 and forms hydrogen bonds to the O2 and O4 of phosphate in chain *B*. This process reinforces the enzyme structure, lowers its flexibility and may improve glucose binding. His160 maintains hydrogen bonds to glucose while forming new interactions with phosphate in chain *A* (Fig. 6 ▸). This dual interaction effectively connects the glucose- and phosphate-binding sites, potentially stabilizing the active site during glucose phosphorylation. These residues also play a critical role in exodomain interactions, stabilizing the enzyme and potentially influencing interactions with other proteins involved in metabolic regulation. Beyond Arg286 and His160, several other structural changes occur upon phosphate binding. Key residues such as those at positions 99–101 (within the β7 sheet), Gly138 (located in the loop before β10) and residues 157–160 (positioned in the loop between β11 and α4) show significant differences when comparing phosphate-bound and phosphate-free structures (Fig. 6 ▸*b*). In the phosphate-free structure, the O atom of Glu187 forms hydrogen bonds to both glucose and water, while in the phosphate-bound complex it forms a new hydrogen bond to Arg286 NH2. This suggests that phosphate indirectly stabilizes glucose binding by modifying hydrogen-bonding patterns and influencing exodomain interactions. Additionally, Ser185 and His183 contribute to these stabilizing effects by forming hydrogen bonds to phosphate, which reduces flexibility and helps to maintain the enzyme conformation during glucose phosphorylation.

Phosphate stabilizes the glucose-binding pocket, primarily through interactions with two key amino acids: His160 and Arg286. His160 forms hydrogen bonds to both phosphate and glucose, linking the phosphate-binding site to the active site and potentially influencing the enzyme activity. Phosphate emerges as a key point of interest due to its abundance in cytosolic conditions, ranging from 1 to 10 m*M* through various homeostatic mechanisms in *E. coli* (Shulman *et al.*, 1979 ▸). The PhoBR two-component signaling system regulates phosphate acquisition and utilization, while excess phosphate leads to polyP accumulation for ATP synthesis (Gardner & McCleary, 2019 ▸). Phosphate may impact enzyme activity and enhance the glucose-utilization efficiency. Additionally, enhanced dynamics at the exodomain suggest that GLK regulation may involve protein–protein interactions with other metabolic regulators or signaling proteins.

## Supplementary Material

PDB reference: *Escherichia coli* glucokinase, 9duc

PDB reference: with glucose bound, 9dvz

## Figures and Tables

**Table 1:** Crystallographic data and refinement statistics for the X-ray structures Values in parentheses are for the highest resolution shell.

	Phosphate–ecGLK	Phosphate–glucose–ecGLK
Data-collection statistics		
Space group	*P*4_3_2_1_2	*P*2_1_2_1_2_1_
*a*, *b*, *c* (Å)	82.11, 82.11, 237.93	47.53, 66.28, 207.53
α, β, γ (°)	90.00, 90.00, 90.00	90.00, 90.00, 90.00
Radiation source	Rigaku PhotonJet-S	Rigaku PhotonJet-S
Wavelength (Å)	1.5418	1.5418
Resolution limits (Å)	29.74–2.63 (2.76–2.63)	22.72–2.54 (2.65–2.54)
*R*_merge_† (within *I*+/*I*−)	0.210 (0.902)	0.202 (0.536)
*R*_merge_ (all *I*+ and *I*−)	0.216 (0.931)	0.218 (0.592)
*R*_meas_‡ (within *I*+/*I*−)	0.233 (1.000)	0.249 (0.657)
*R*_meas_ (all *I*+ and *I*−)	0.228 (0.980)	0.243 (0.657)
*R*_p.i.m._§ (within *I*+/*I*−)	0.100 (0.425)	0.143 (0.375)
*R*_p.i.m._ (all *I*+ and *I*−)	0.072 (0.304)	0.107 (0.282)
*R*_merge_ in top intensity bin	0.077 (—)	0.073 (—)
No. of observations	247590 (35037)	111209 (14258)
No. of possible unique observations	25106 (3266)	22512 (2708)
No. of unique observations	25056 (3266)	22445 (2708)
Mean *I*/σ(*I*)	9.2 (3.7)	5.5 (2.6)
CC_1/2_	0.991 (0.743)	0.983 (0.802)
Completeness (%)	99.8 (100)	99.7 (100)
Multiplicity	9.9 (10.7)	5 (5.3)
Mean χ^2^	0.95 (0.93)	0.92 (0.96)
Refinement statistics		
Resolution (Å)	29.74–2.63	22.72–2.54
Protein residues	638	638
Solvent molecules	173	176
Phosphate molecules	2	2
*R*_free_	0.230	0.257
*R*_work_	0.191	0.205
R.m.s.d.		
Bond lengths (Å)	0.002	0.002
Angles (°)	0.507	0.554
Ramachandran plot		
No. of residues	638	638
Most favored regions (%)	93.71	92.87
Allowed regions (%)	6.29	7.13
Mean *B* factors (Å^2^)		
Protein	33.4	23.1
Phosphate	30.0	11.5
Glucose	—	12.9

† *R*_merge_, also known as *R*_sym_, is a discrepancy index used to measure the equivalence or internal consistency among intensities of equivalent reflections in X-ray crystallo­graphy.

‡ *R*_meas_ is a discrepancy index introduced to account for the dependence of *R*_merge_ (*R*_sym_) on the multiplicity, which is the number of equivalent reflections.

§ *R*_p.i.m._, or the precision-indicating merging *R* factor, is a discrepancy index introduced to further refine the dependence of *R*_meas_ on the multiplicity.

## Data Availability

All data created during this research are openly available from the Protein Data Bank under the provided accession numbers. This study includes the re-analysis of existing data, which are openly available at the locations cited in the references.
